# Cinnamaldehyde improves methamphetamine-induced spatial learning and memory deficits and restores ERK signaling in the rat prefrontal cortex

**DOI:** 10.22038/IJBMS.2018.35368.8427

**Published:** 2018-12

**Authors:** Mohammad Saeed, Ameneh Ghadiri, Farzin Hadizadeh, Armin Attaranzadeh, Mohaddeseh Sadat Alavi, Leila Etemad

**Affiliations:** 1 School of Pharmacy, Mashhad University of Medical Sciences, Mashhad, Iran; 2 Department of Internal Medicine and Medical Specialties, Sapienza University of Rome, Rome, Italy; 3 Biotechnology Research Center, Pharmaceutical Technology Institute, Mashhad University of Medical Sciences, Mashhad, Iran; 4 Department of Medicinal Chemistry, School of Pharmacy, Mashhad University of Medical Sciences, Mashhad, Iran; 5 Milad Infertility Center, Imam Reza Hospital, Mashhad University of Medical Sciences, Mashhad, Iran; 6 Division of Neurocognitive Sciences, Psychiatry and Behavioral Sciences Research Center, Mashhad University of Medical Sciences, Mashhad, Iran; 7 Pharmaceutical Research Center, Pharmaceutical Technology Institute, Mashhad University of Medical Sciences, Mashhad, Iran

**Keywords:** Cinnamaldehyde, ERK1/2, Learning deficit, Memory deficit, Methamphetamine

## Abstract

**Objective(s):**

Methamphetamine is a stimulant compound that penetrates readily into the central nervous system. Repeated exposure to methamphetamine leads to damage in the dopaminergic and serotonergic axons of selected brain regions. Previous studies showed that cinnamaldehyde improved memory impairment in animals. In the present study, we aimed to elucidate the effects of cinnamaldehyde on methamphetamine-induced memory impairment in rats.

**Materials and Methods:**

Male Wistar rats received methamphetamine (10 mg/kg, intraperitoneally) for 7 days. Thirty minutes before each injection, animals were given cinnamaldehyde (20, 40, or 80 mg/kg) or rivastigmine (1 mg/kg). The spatial learning and memory were examined using the Morris water maze test. The expression of extracellular signal-regulated kinase (ERK) phosphorylation in the frontal cortex and hippocampus was also detected by immunohistochemical method.

**Results:**

Administration of methamphetamine increased the latency to find the platform in the learning phase, while administration of cinnamaldehyde (40 mg/kg) or rivastigmine before methamphetamine reversed the increased latency. Administration of cinnamaldehyde, at the dose of 40 mg/kg with methamphetamine, increased the time and distance traveled in the target quadrant in comparison with the amphetamine group. Moreover, the methamphetamine and cinnamaldehyde-treated group had higher expression of phosphorylated ERK1/2 in the prefrontal cortex in comparison with the methamphetamine-treated animals.

**Conclusion:**

The present data demonstrated that repeated METH administration impaired cognitive performance through the ERK pathway and decreased the phosphorylation of ERK1/2 in the prefrontal cortex while administration of cinnamaldehyde restored both effects. Accordingly, cinnamaldehyde may be a valuable therapeutic tool for the treatment of cognitive deficits associated with methamphetamine consumption.

## Introduction

Methamphetamine (METH) or crystal METH is a powerful stimulant drug that belongs to the amphetamine family. According to the published data, METH consumption is ranked as the second drug abused all over the world (1). Probably, low cost of synthesis and relative ease of access are the main reasons for the rise in METH abuse. METH has also been approved by the U.S. Food and Drug Administration (FDA) for treatment of obesity and the attention deficit hyperactivity disorder (ADHD) in children(2). Acute and chronic administration of METH produces a range of adverse effects including mental, behavioral, and cardiovascular disorders. METH can easily cross the blood-brain barrier and penetrate into the central nervous system (CNS) (3, 4). METH enhances the release of norepinephrine, serotonin, and dopamine from the cytosol into the synapses. In fact, it acts as an indirect agonist of monoamine receptors (5). Acute and especially long-term exposure to METH leads to neurological deficits through chemical and molecular changes including excitotoxicity, oxidative stress, and apoptosis (6). Repeated exposure to METH impairs spatial learning and memory. METH exposure, even after many years of abstinence, produces deficits in selective attention, working memory, impulse control and information processing. It is well documented that extracellular signal-regulated kinase (ERK) pathway is involved in METH- induced behavioral and cognitive responses. ERK, a member of the mitogen-activated protein kinase (MAPK) superfamily, is expressed widely in the PFC, hippocampus and striatum. The activated ERK, phosphorylated ERK (pERK), activates downstream of cAMP-response element-binding protein (CREB) and exerts broad effects on biological and behavioral responses (7, 8).

*Cinnamomum cassia* (*C. cassia*) is a traditional medicinal herb that has been used worldwide especially in Asia to treat diabetes, dyspepsia, anxiety, ischemia, cancers, and inflammatory diseases (9-12). Cinnamaldehyde, also known as cinnamic aldehyde (CA), is a natural product from cinnamon tree, which is a yellow viscous liquid (13). It is the main component of cinnamon oil produced from the stem bark of *C.cassia *(14). CA has a broad range of biological effects including anti-tumor (15), antioxidant , and anti-inflammatory activities (16). In addition, CA plays neuroprotective roles in Alzheimer’s and Parkinson’s diseases while its mechanism(s) of action is as yet unclear (17, 18). 

Considering the potential neuroprotective properties of CA, this study was designed to elucidate the effects of CA on learning and spatial memory deficits induced by METH in rats and to explore the underlying mechanism.

## Materials and Method

***Animals and drugs***

Seven groups of six male Wistar rats, weighing 200–250 g, were obtained from the Animal Center, Pharmaceutical Technology Institute, Mashhad University of Medical Sciences. The animal house temperature was maintained at 23±2 ^°^C with a 12 hr light/dark cycle. Rats had free access to food and tap water. Experiments were approved by the Animal Care Committee of Mashhad University of Medical Sciences, Mashhad, Iran. 

CA and rivastigmine were purchased from Sigma (Germany). METH was donated by the Department of Medicinal Chemistry, School of Pharmacy, Mashhad University of Medical Sciences (Mashhad, Iran). Cinnamaldehyde was dissolved in 1% Tween 80 plus sterile normal saline as vehicle (19). All other chemicals were dissolved in normal saline. The anti-phospho-P44/42 Map (Erk1/2) and rabbit polyclonal antibody were purchased from Cell Signaling Technology (#9101 and #7074).

**Figure 1:**
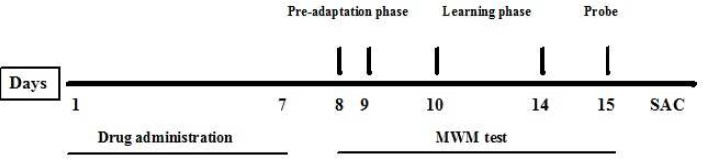
Diagrammatic sketch of the behavioral and histopathological experiments. MWM: Morris water maze; SAC: sacrificed for histopathological experiments

**Figure 2:**
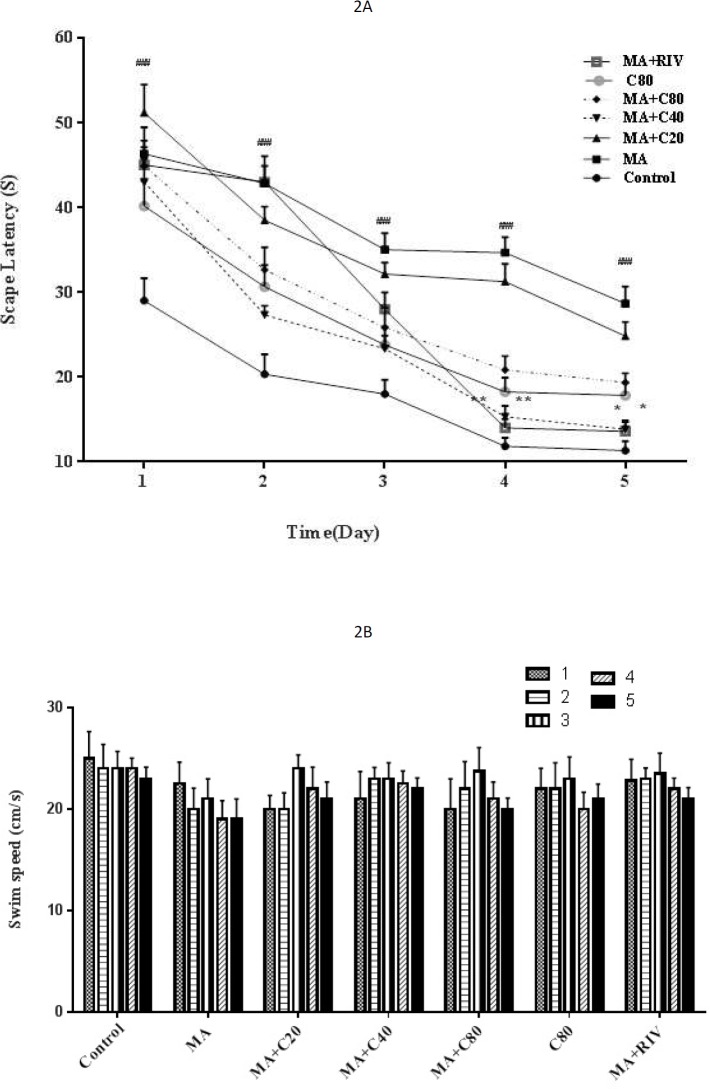
Escape latency (A) and swim speed (B) in METH-treated rats (10 mg/kg) that received CV (20, 40, or 80 mg/kg) or RIV (1 mg/kg) in the training days using the Morris water maze test. METH: Methamphetamine, CA: Cinnamaldehyde, RIV: Rivastigmine. The results are reported as mean+SEM, ### *P<*0.001 compared with the control group; * *P<*0.05, ** *P<*0.01 compared with the METH group

**Figure 3:**
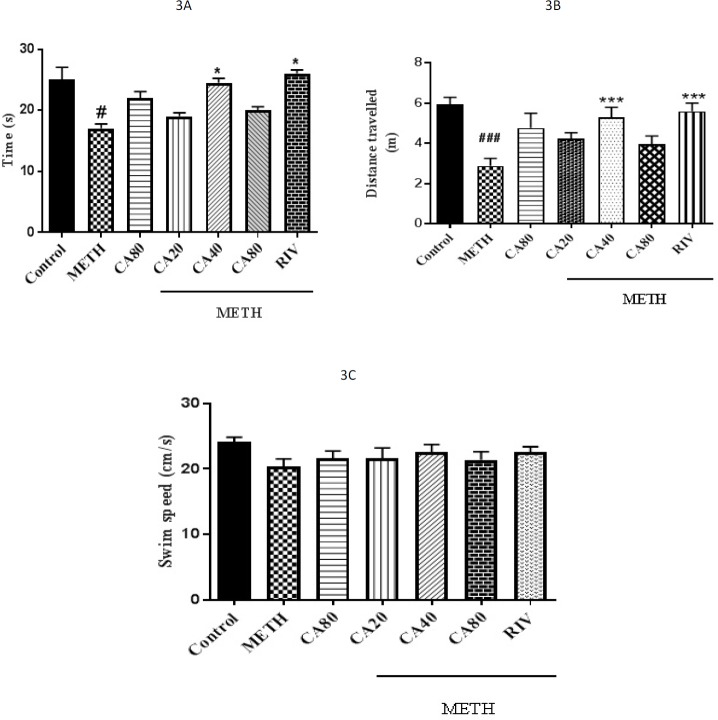
Time spent (A), distance traveled (B), and swim speed (C) in METH-treated rats (10 mg/kg) that received CV (20, 40, or 80 mg/kg) or RIV (1 mg/kg) in the target quadrant using the Morris water maze test. METH: Methamphetamine, CA: Cinnamaldehyde, RIV: Rivastigmine. The results are reported as mean+SEM, # *P<*0.05 and ### *P<*0.001 compared with the control group; * *P<*0.05, *** *P<*0.001 compared with the METH group

**Figure 4:**
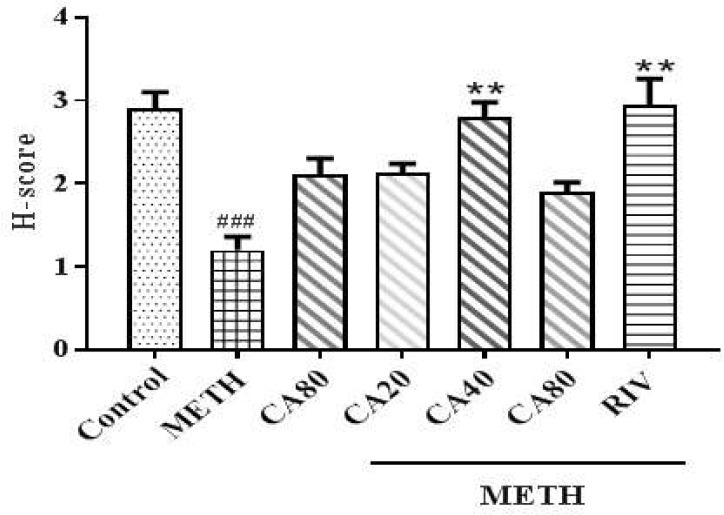
Effects of methamphetamine, cinnamaldehyde, and rivastigmine on phospho ERK levels using H-Score. METH: Methamphetamine, CA: Cinnamaldehyde, RIV: Rivastigmine. ### *P< *0.001 compared with the control group and ** *P<* 0.01 compared with the METH group

**Figure 5:**
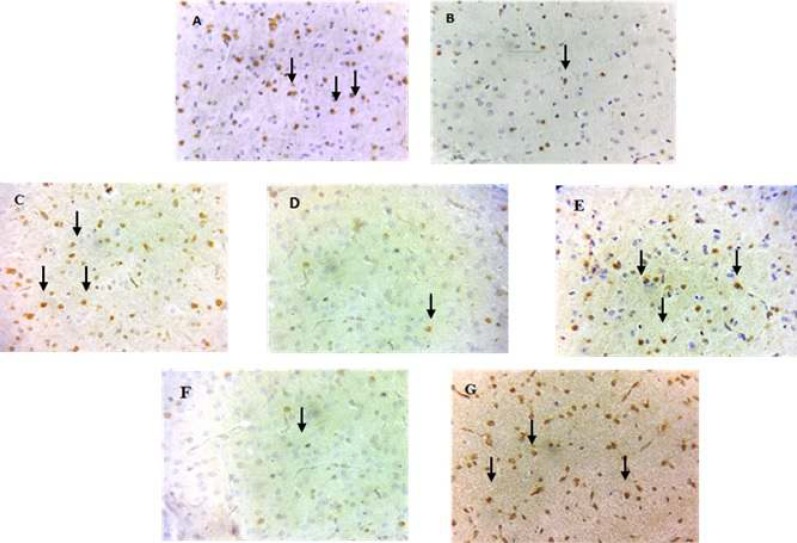
Phospho-ERK 1/2 expression in the prefrontal cortex of rats that received normal saline plus tween (A), METH (B), CA (80 mg/kg) (C), METH plus CA (20, 40, or 80 mg/kg) (D, E, and F, respectively) or METH plus RIV(G). Phospho-ERK 1/2-positive cells were decreased after METH exposure and reversed after RIV and CA (40 mg/kg) administrations. METH: Methamphetamine, CA: Cinnamaldehyde, RIV: Rivastigmine. Magnification = 400X

***Groups and treatments***

Five groups of rats were treated by METH (10 mg/kg) for seven days. Thirty min before METH injection, they received vehicle (control group), CA (20, 40 and 80 mg/kg) or rivastigmine (RIV) (1 mg/kg) (positive control group). Controls received normal saline and one group received normal saline plus CA at the dose of 80 mg/kg. All injections were given intraperitoneally (IP). At the end of the seventh day, spatial learning and memory were examined using the Morris water maze (MWM) test. One day after completion of the MWM test, all animals were sacrificed and their brains were dissected and stored in formalin (Figure 1).

***Morris water maze***

The Morris water maze consisted of a black circular tank (160 cm in diameter and 60 cm high) filled with water (depth 25 cm, 20–22 ^°^C) and a square platform (10 cm diameter) was submerged 2 cm below the surface of the water in the center of the west quadrant. The pool was divided into four equal size quadrants, as four different starting points, labeled North (N), South (S), East (E), and West (W). The MWM test was performed in three distinct phases. In brief, at the pre-adaptation phase, each rat was habituated to MWM without the platform for 30 sec. During the acquisition phase, the rats were allowed a maximal time of 60 sec for swimming to find the hidden platform (escape latency) with a resting time of 15 sec on the platform. For each animal, four trials were carried out daily for 5 consecutive days. In the probe test as the third phase, the platform was removed and the animals were allowed to swim for 60 sec. The swim speed, time spent in the target quadrant, and distance traveled in the target quadrant, where the platform was previously found, were recorded using a camera above the pool and analyzed using a software package (Radiab, Ver 2.1, Iran) (20).

***Immunohistochemical evaluation***

After completion of the MWM task, rats were sacrificed and the brains were removed and placed into 10% formalin and processed for paraffin embedding. The blocks were cut at 5 μm thickness. For the histopathological examination, tissue sections were placed on slides and dried overnight at 45 °C, dewaxed in xylene, and rehydrated in graded alcohols. Endogenous peroxidase activity was blocked by incubation in 3% hydrogen peroxide (H_2_O_2_) and then incubated in a blocking solution. The primary antibody, phosphorylated ERK1/2, was placed on the slides at appropriate dilution. Sections were then incubated with rabbit anti-IgG conjugated to horseradish peroxides (Dako) and DAB substrate. 

The phosphorylated ERK1/2 immunoreactivity level of each sample was evaluated semi-quantitatively under a light microscope by evaluating the signal intensity (0, 1+, 2+, or 3+) and the percentage of cells showing positive nuclear staining (0, none; 0.1, less than one-tenth; 0.5, less than one half; and 1, greater than one half). The intensity and proportion scores were then multiplied using a semi-quantitative scoring method to assign a histochemical score (H-score)(21). 

***Statistical analysis***

All data were expressed as mean±SEM. In the Morris water maze test, the swimming speed and time spent to find the platform during training sessions were analyzed using repeated measures ANOVA. The time spent, distance traveled, and swimming speed in the probe trial were analyzed using one-way ANOVA followed by Tukey’s test. In order to compare the mean H-Score, Kruskal–Wallis test followed by unpaired Mann-Whitney post hoc test was performed. *P<* 0.05 was considered as the level of statistical significance.

## Results

***Effect of METH and CA on escape latency and swim speed during the learning phase***

The results showed that during the acquisition phase, the performance of all groups was improved as indicated by decreased escape latency. However, administration of METH increased latency to find the hidden platform during five days compared with the control group (*P<* 0.001). RIV and CA (40 mg/kg) when administrated before METH, decreased latency time, which was significantly different from the METH-treated group at days 4 and 5 (*P<* 0.01 and *P<* 0.05, respectively) (Figure 2A). There were no differences between the swimming speeds of any group during the learning phase (Figure 2B).

***Effect of METH and CA on time spent, distance traveled, and swim speed in the probe trial***

The results showed that METH-treated rats failed to remember the platform location such that the time spent and distance traveled in the target quadrant were decreased (*P<*0.05 and *P<*0.001, respectively). Treatment with CA (40 mg/kg) and RIV significantly increased the time spent in the target quadrant, as compared with the METH group (Figure 3A, *P<*0.05). In the same way, as shown in figure 3B, the distance traveled in the target quadrant was significantly increased following co-administration of CA (40 mg/kg) and RIV with METH compared with the METH group, implying improvement of memory recall. Furthermore, there were no significant differences between the swimming speeds of any group on the 15^th^ day of the experiment (Figure 3C).

***Effect of METH and CA on PFC and hippocampal phospho-ERK1/2 levels***

The results of PFC immunohistochemistry staining in different treatment groups are shown in Figure 4. The METH group exhibited a lower phospho-ERK1/2 level than the control group (*P<*0.001). Co-administration of CA (40 mg/kg) and METH increased the phospho-ERK1/2 level compared with the METH group (Figure 4, *P<*0.01). The riv-treated group had also an increased phospho-ERK1/2 level compared with the METH group (Figure 5, *P<*0.01). There were no changes in the phospho-ERK1/2 levels in the hippocampus of any group (data not shown).

## Discussion

METH is a powerful mental stimulant that is widely abused. Scientists believe that long-term METH use leads to cognitive defect in humans. Clinically, consumption of this substance has been associated with various disorders such as verbal and nonverbal memory impairment, attention deficit disorder, and motor impairment (22, 23). Morris water maze has been introduced as a valid animal model to investigate spatial learning and memory in rodents (24). The results of this study showed that CA at the dose of 40 mg/kg and RIV when co-administrated with METH significantly reversed the METH-induced memory impairment by increasing the time and distannced travel in the target quadrant in probe trial. To evaluate the influence of swimming ability on learning performance, mean speeds of all animals were measured and no significant difference was found between groups indicating that motor function and motivational and visual abilities were not affected during experiments. It was reported that pretreatment with Cinnamon extract (200 and 400 mg/kg, orally, 21 days) significantly reversed the scopolamine deteriorative effect on escape latency in the MWM test (25). 

Cognitive impairment during chronic administration of METH has been attributed to changes in the ERK signaling pathways (8). ERK belongs to MAPKs family and plays a critical regulatory role in cellular functions such as differentiation, proliferation, development, as well as, memory and learning processing. Accumulating evidence indicates that ERK cascade is implicated in learning and formation of memory and behavioral responses to drugs of abuse such as METH and cocaine (8). The present data showed that repeated METH administration impaired cognitive performance through the ERK pathway and decreased the phosphorylation of ERK1/2 in the PFC, while co-administration of CA and RIV restored it. However, ERK expression in the hippocampus was not affected by METH administration. In accordance with our study, it has been reported that repeated METH treatment increased escape latency in the training phase and decreased the number of crossings of the target quadrant in the probe test and modulated the MAPK-ERK signaling pathway by reduction of the ERK1/2 level in the PFC but not in the hippocampus and led to memory and learning abnormalities (7). The results of other studies also revealed that METH administration in single dose can induce apoptosis and gliosis in striatum and cortex regions and reduction in phospho ERK in PFC at repeated dose in mice (26, 27). The PFC has an essential role in short-term/working memory and decision-making, while the hippocampus is involved in the formation of long-term memories. The PFC mediates decision-making functions and is, therefore, a key neuroanatomical region in addictive behaviors (7). 

Evidence suggests that cinnamon has a potential role in the prevention of neurodegenerative diseases (28). The protective effect of cinnamon and its active components against neuroinflammation was evaluated using lipopolysaccharide (LPS)-induced inflammation both *in vitro* and *in vivo *(28). In a mouse model of LPS-induced memory impairment and synaptic plasticity inhibition, CA pretreatment decreased nitric oxide production and interleukin-1β release in primary microglia. CA was also able to reduce the levels of phosphorylated ERK1/2 in the hippocampus of mice treated with LPS and interfered with the MEK1/2-ERK1/2 signaling pathway and destabilized inducible nitric oxide synthase mRNA expression. CA significantly diminished memory deficit and improved synaptic plasticity in LPS-treated mice (29). Cinnamon and its metabolite, sodium benzoate, can activate the cAMP response element binding protein (CREB) via protein kinase A (PKA) and up-regulation of molecules associated with hippocampal plasticity. In agreement, oral administration of cinnamon and sodium benzoate improved spatial memory consolidation and transformed poor learning mice to good learners (30).

 Based on our findings, METH treatment induced neuronal abnormalities in the PFC, but not in the hippocampus, and CA at medium dose significantly reversed it. The results of our study showed that CA at the dose of 80 mg/kg did not improve memory impairment or the expression level of phosphorylated ERK1/2. This finding is in accordance with the results of Iersel *et al.* They investigated the effect of CA on glutathione S-transferase activity in skin cancer cells. CA increased the level of GSH against the carbonyl derivatives toxicity in human melanoma cells. However, this effect was reversed at concentrations higher than 40 μM (31). This finding implies that the protective effects of CA may be vanished at certain doses and this should be considered in future studies.

## Conclusion

The study data showed that cinnamaldehyde had neuroprotective effects and improved cognition and learning function in METH-treated animals through activation of the ERK pathway in the PFC. Our findings also showed that the effects of CA were not dose-dependent and this may limit the clinical applications of this natural compound. 
